# Nutrients in the Prevention of Alzheimer's Disease

**DOI:** 10.1155/2019/9874159

**Published:** 2019-09-04

**Authors:** Anna Laura Cremonini, Irene Caffa, Michele Cea, Alessio Nencioni, Patrizio Odetti, Fiammetta Monacelli

**Affiliations:** Department of Internal Medicine and Medical Specialties, Section of Geriatrics, IRCCS Ospedale Policlinico San Martino, Genova, Italy

## Abstract

Alzheimer's disease (AD) is a disease caused by the complex interaction of multiple mechanisms, some of which are still not fully understood. To date, pharmacological treatments and supplementation of individual nutrients have been poorly effective in terms of the prevention and treatment of AD, while alternative strategies based on multimodal approaches (diet, exercise, and cognitive training) seem to be more promising. In this context, the focus on dietary patterns rather than on single food components could be more useful in preventing or counteracting the pathological processes typical of AD, thanks to the potential synergistic effects of various nutrients (neuronutrients). The aim of this narrative review is to summarize the currently existing preclinical and clinical evidence regarding the Mediterranean diet (MeDi), the Dietary Approaches to Stop Hypertension (DASH) diet, and the Mediterranean-DASH Intervention for Neurodegenerative Delay (MIND) diet, which are three dietary patterns with well-known anti-inflammatory and antioxidant properties. Recently, they have been related to brain protection and AD prevention, perhaps thanks to their high content of neuroprotective bioactive compounds. Similarly, intermittent fasting (IF) or calorie restriction (CR) is emerging as interesting approaches that seem to promote hippocampal neurogenesis, activate adaptive stress response systems, and enhance neuronal plasticity, thus leading to motor and cognitive improvements in animal models of AD and hopefully also in human beings.

## 1. Introduction

Alzheimer's disease (AD) is a disease of later life, affecting one in four people 85 years of age or over, and the incidence is expected to rise in the coming years, with 131.5 million estimated cases by 2050 [1]. To date, approved drugs have shown modest clinical benefits in delaying the neurodegenerative process, and currently, the AD epidemic is facing two major challenges. Both the lack of disease modifying drugs and the need to delay cognitive-related disability and frailty trajectories highlight the necessity to develop nonpharmacological strategies to stop this ever-increasing global burden. In line with this, previous studies, including the milestone Rotterdam study, have pointed out the relevance of nutrition in counteracting brain neurodegeneration [2].

Although several nutritional approaches have been considered as possible alternatives to the currently existing drugs for AD, this line of research has only been partially explored and has not resulted in any solid evidence [3, 4].

The neuropathology and etiology of AD indicate that a complex series of molecular mechanisms is involved, including the amyloid hypothesis, mitochondrial dysfunction, oxidative stress, and brain neuroinflammation as the mainstream molecular pathways [5]. In turn, the neuroinflammatory cascade may be synergistically associated with immunosenescence and gut dysbiosis, intercepting the trajectories of the aging brain and the progression to dementia [6].

Recent evidence has indicated that epigenetics could help shed light on such a complex neurodegenerative pattern. DNA methylation, histone modifications, and microRNAs are the principal epigenetic mechanisms involved in AD pathophysiology. In line with this, nutrition is believed to be a modifiable environmental factor that seems to strongly impact on AD pathology by modulating its phenotypic expression [7, 8].

Therefore, recent literature reports have underlined the protective role of a number of individual food components, including micro- and macronutrients in the prevention and management of AD [9, 10]. Several researchers have explored the role of single food components, as well as lifestyle habits and inappropriate diets in facilitating the development of AD and its clinical progression. By virtue of the role of cardiovascular risk factors in the onset of AD [11], nutritional approaches targeting insulin resistance, dyslipidemia, and oxidative stress have been found to ameliorate the related clinical conditions, such as diabetes, metabolic syndrome, and dyslipidemia [12–14].

In keeping with the understanding of the complex interplay between nutrition and AD, a multinutrient approach has also been developed which is based on the rationale that multiple dietary molecules can interact in a synergistic manner to modulate several AD molecular hallmarks.

Namely, omega-3 fatty acids, vitamin B and E, choline, and uridine have provided the rationale for improving effectiveness in AD prevention and clinical management. However, to date, no clinical evidence that this putative nutritional supplementation prevents AD onset or progression has been reported [15]. Based on the current findings, it is unlikely that a single food component or a multinutrient supplementation actually represents the right way to prevent the development of AD or slow down its progression.

However, the interactions among several individual nutrients seem to provide the ground for effectiveness with respect to dementia prevention in older adults.

Another important aspect is that all the theoretical evidence that has been collected so far shows a series of pitfalls that hamper practical concepts and the clinical transferability of results.

The main limitations that prevent bench to bedside translation may be related to the experimental conditions and the pharmacological concentrations of the specific food components that hardly mimic human dietary intake and daily recommended doses. Although animal studies seem to be promising, few and contradictory results have been observed in human trials [16]. Moreover, the heterogeneity of the study designs and the paucity of large scale clinical epidemiological and observational studies on the causal link between nutrition and AD make the results even more difficult to understand.

Besides these issues, strategies that focus on dietary patterns rather than on an approach based on individual foods or nutrients seem to provide a unifying conceptual framework between nutrition and AD; the various components or “neuronutrients” included in a good dietary pattern can offer potential synergistic and neuroprotective effects [16–18].

Adopting this as our starting point, our review will summarize the latest developments regarding the use of dietary patterns in older adults as a way to prevent AD.

Thus, studies whose outcomes include “cognitive functions” or “global cognitive performance” or the incidence of a generic “cognitive decline” or “cognitive impairment” will not be taken into consideration.

## 2. Mediterranean Diet (MeDi) and AD Prevention

The Mediterranean diet (MeDi) would appear to be promising for AD prevention, including the earlier predementia stages. Indeed, the MeDi diet, based on traditional eating habits in Greece, Southern Italy, and other Mediterranean regions, albeit with regional differences, is characterized by high consumption of fruits and vegetables, cereals, legumes, olive oil, nuts, and seeds as the major source of fats, moderate consumption of fish, low to moderate consumption of dairy products and alcohol (wine), and low intake of red and processed meats (see Table 1). It can be considered a nutritional model for healthy dietary habits since it contains all the essential nutrients including monounsaturated fatty acids (mainly in olive oil), polyunsaturated fatty acids (in fatty fish), antioxidants (e.g., allium sulphur compounds, anthocyanins, beta-carotene-flavonoids, catechins, carotenoids, indoles, or lutein), vitamins (A, B1, 6, 9, 12, D, and E), and minerals (magnesium, potassium, calcium, iodine, zinc, and selenium) [19]. Growing evidence indicates the neuroprotective potential of the MeDi, thus supporting the rationale that adherence to this dietary pattern can be a preventative approach towards reducing the risk of cognitive decline, mild cognitive impairment (MCI), and AD [20, 21].

### 2.1. Epidemiological Evidence (See Table 2)

Two cross-sectional studies [22, 23] showed an inverse correlation between the Mediterranean diet and AD in older American and Australian adults. In the first study [22], which was performed on a cohort of elderly American subjects living in New York, the MeDi score (which is a 9-point scale developed on the basis of the eating habits of a Greek population, with higher scores indicating greater adherence) was the main predictor of AD status in logistic regression models adjusted for potential confounders for both AD (age, sex, ethnicity, education, apolipoprotein E genotype, caloric intake, smoking, medical comorbidity index, and body mass index) and for vascular risk factors (dyslipidemia, hypertension, and coronary heart disease) that should be considered possible mediators in the pathogenesis of AD. Higher adherence to the MeDi was associated with a significantly lower risk of AD, considering MeDi adherence both as a continuous and a categorical variable [24]. In the second cross-sectional study, Gardener et al. [23] replicated these results in an Australian population of older adults participating in the Australian Imaging, Biomarkers and Lifestyle Study of Ageing (AIBL) study. As compared to healthy controls, subjects with a diagnosis of MCI or AD had a lower mean MeDi score, and every 1-unit increase in the MeDi score was associated with a 19–26% decrease in the odds of being in the AD category.

Several prospective studies have been published in the last 15 years examining the role of the MeDi diet in reducing the risk of dementia and AD. Numerous US population-based studies having a median follow-up of 3–5.4 years revealed that greater adherence to the MeDi was associated with a reduced risk of AD [25–28], a lower risk of developing AD in patients with MCI [29], and lower mortality in AD patients, suggesting a possible role of MeDi in modulating not only the pathogenetic pathways but also the subsequent course of AD [30]. A more recent longitudinal study conducted in a Greek population as part of the Hellenic Longitudinal Investigation of Ageing and Diet (HELIAD) [31] evaluated adherence to the MeDi pattern using a more complicated score, i.e., the Mediterranean Dietary Score (MedDietScore) [32]. The authors of the HELIAD study found that each unit increase in the MedDietScore was associated with a 10% decrease in the odds of dementia.

Unlike the previously mentioned studies, three other prospective studies found no association. In a French study, MeDi adherence was not associated with a risk of incident dementia or AD as a continuous or as a categorical variable [33]. In another study, Roberts et al. reported a 25% reduced risk of MCI or dementia in subjects in the upper tertile of the MeDi score at baseline, but this association did not reach statistical significance, possibly due to the relatively short follow-up (median follow − up = 2.2 years) [34]. In the third one, Olsson et al. found no correlation between MeDi adherence and the risk of AD or all-type dementia in a cohort of 1,138 elderly Swedish men followed-up for 12 years [35].

Several systematic reviews and meta-analyses of both case-control and longitudinal studies confirmed the association between higher adherence to MeDi and a reduced risk of stroke, depression, and neurodegenerative diseases (cognitive decline, dementia, MCI, AD, and Parkinson's disease), albeit with some contradictory results [36–41]. Several factors can at least partly explain these differences in results. The first one is the use of different methods for evaluating eating habits (0 to 9 score, 0 to 55 score, and others): these scores are usually validated in a specific population having specific characteristics, eating habits, and culture. Therefore, they cannot easily be applied to other populations, especially non-Mediterranean ones, such as the Americans or Australians. As already discussed with regard to the assessment of MeDi adherence, the two most commonly used scores are the Trichopoulou's 0 to 9 score [24] and the Panagiotakos's 0 to 55 score [32]. The use of these two scoring systems has been extensively reported in the literature and both have proven to be reliable and valid tools for assessing adherence to the Mediterranean diet, but they are both based on the typical eating habits of the Greek population, so it is difficult to apply these scores to non-Mediterranean populations. Moreover, there is broad heterogeneity in the study characteristics, such as the mean age of subjects, the duration of follow-up, and the high number of neuropsychological tests used for the diagnosis of MCI and/or AD.

### 2.2. Randomized Controlled Trials (RCTs)

Only few RCTs (PREDIMED, MedLey, and NU-AGE) have assessed the effects of a Mediterranean dietary pattern on cognition in older adults both in Mediterranean and non-Mediterranean countries (see Table 2).

The first RCT to evaluate the effects of long-term MeDi intervention on cognitive function and to shed some light on the role of dietary patterns in counteracting the neurodegenerative process was carried out on a subcohort of the well-known multicenter PREDIMED trial, which was a milestone in establishing the strong preventive role of the MeDi in individuals at a high cardiovascular risk [42]. The nutritional intervention of PREDIMED consisted in a typical MeDi supplemented with extravirgin olive oil or mixed nuts (foods with antioxidant and anti-inflammatory properties) compared to a control low-fat diet. Martinez-Lapiscina and colleagues [43] enrolled 522 subjects and assessed the overall cognitive performance at study completion alone, after 6.5 years. They reported a significant difference in mean Mini-Mental State Examination (MMSE) and Clock Drawing Test (CDT) scores in both intervention groups versus the low-fat control group. There were two main limitations in this study: (1) the lack of evaluation of cognitive performance at the beginning of the study, which did not allow to evaluate changes in cognitive functions over time and (2) the insufficient statistical power to demonstrate a protective effect of MeDi on dementia development, given the small number of total incident cases that were observed during the follow-up.

After a few years, Valls-Pedret et al. [44] published the first MeDi intervention trial demonstrating a positive effect on cognition of the MeDi supplemented with either nuts or extravirgin olive oil. In this PREDIMED substudy, cognitive performance was evaluated both pre- and postintervention, thus enabling the researchers to detect any significant cognitive improvement in the participants allocated to the MeDi intervention groups, who were assessed after a median of 4.1 years.

The MedLey study was the first RCT conducted in older non-Mediterranean adults. A total of 137 subjects were randomly assigned to either a MeDi or a control diet (their usual diet) for six months. This study did not find any significant beneficial effects of a MeDi intervention on cognitive functions (executive functioning, speed of processing, memory visual-spatial ability, and overall age-related cognitive performance) among healthy older adults, perhaps because of the short duration of the intervention, the relatively limited number of participants, or the “Australianization” of the MeDi (i.e., based on the Australian foods and habits, rather than on the traditional MeDi), resulting in smaller nutritional intervention differences between the intervention groups and the control group [45].

The NU-AGE trial (NCT01754012), a recent 1-year multicenter RCT, was carried out in both Mediterranean (including Italy) and non-Mediterranean European countries and allocated a total of 1,279 healthy older adults to two parallel groups. The intervention group followed the NU-AGE diet, which consisted of a culturally adapted and individually tailored Mediterranean-like diet on the basis of the specific dietary recommendations for older adults in the various countries involved in the study. One year later, at the end of the study, all participants showed improvements in their cognitive performance but the differences between the two groups did not reach statistical significance [46]. Nonetheless, the authors highlighted that the participants in the intervention group with the highest adherence to the NU-AGE diet showed a significant improvement in episodic memory, the impairment of which is a core feature of AD [47].

To date, results from RCTs are mostly nonsignificant, with small effect sizes and little or no benefit of the MeDi for incident cognitive impairment or dementia, as also documented by a recent systematic review of RCTs by Radd-Vagenas et al. [48]. RCTs with a longer duration and higher number of participants are needed to establish whether adherence to the MeDi can help prevent (or delay) the onset of AD and dementia.

### 2.3. Mediterranean Diet and Brain Imaging

AD is a neurodegenerative disease characterized by typical changes in the brain that can be detected by a variety of imaging modalities, including structural and functional Magnetic Resonance Imaging (MRI) and Positron Emission Tomography (PET). In recent years, cross-sectional and longitudinal studies have shown the relationship between lifestyle and dietary habits and the morphological, structural, functional, and metabolic modifications of the brain regions that undergo neurodegeneration in the preclinical and clinical phases of AD.

#### 2.3.1. Mediterranean Diet and Structural Brain Modifications

Growing evidence, derived mainly from MRI studies, suggests an association between higher MeDi adherence and preservation of brain structures, in particular in the so-called “Regions-of-Interest” (ROIs) which are vulnerable to the neurodegenerative changes typical of AD. Higher MeDi adherence was associated with a significantly greater thickness of 3 ROIs (orbitofrontal cortex, entorhinal cortex, and posterior cingulate cortex of the left hemisphere) in a cross-sectional study performed on an American population of cognitively normal middle-aged participants [49]. Similar findings were reported by two other cross-sectional American studies in which the greatest benefit in terms of brain structure preservation was attributed to the higher intake of fish and legumes and lower intake of meat [50, 51]. More recently, Karstens et al. found that higher adherence to the Mediterranean pattern was associated with better learning and memory performance and larger bilateral dentate gyrus volumes after adjusting for a number of confounding factors [52].

Conversely, several longitudinal studies reported a negative effect on brain structures related to lower adherence to the MeDi [53] or adherence to an unhealthy Western dietary pattern [54], while a Swedish longitudinal study including 194 cognitively healthy elderly individuals found no association between the total MeDi score and the brain volumes perhaps due to the intrinsic limitations of the study design [55].

In conclusion, gray matter atrophy and reduced cortical thickness in the medial temporal lobe are a typical early feature of AD, and the association of MeDi adherence with greater gray matter volumes in AD regions may indicate some direct involvement of this type of dietary pattern in preventing or slowing the neurodegeneration and the consequent neuronal loss that is typical of this disease. More recently, Pelletier and colleagues reported a positive association between higher MeDi adherence and a general pattern of preserved white matter microstructure with no relation to gray matter volumes, suggestive of alternative mechanisms partly independent of AD-related neurodegeneration, possibly including vascular pathways [56]. The beneficial effect of the MeDi on vascular risk factors (e.g., lipid profile, blood pressure, insulin resistance, adiposity, inflammation, and oxidative stress) is in fact well established [57–60].

#### 2.3.2. Mediterranean Diet and Functional Brain Modifications

The AD brain is characterized by several metabolic changes that can also be found in other neurodegenerative diseases and in normally aging brains and represent nonspecific biomarkers of impairment of neuronal activity and synaptic transmission. These metabolic and functional modifications can be detected by PET, in particular, fluorodeoxyglucose- (FDG-) PET and Pittsburgh Compound B- (PiB-) PET which allow us to evaluate changes in cerebral glucose metabolism (an indicator of neuronal network activity) and the degree of beta-amyloid (A*β*) deposition in brain regions known to be involved in AD. Studies concerning the association between MeDi adherence (and in general, adherence to a healthy dietary pattern) and functional neuroimaging outcomes are limited.

Two cross-sectional studies in the American population found an association between higher adherence to a MeDi pattern and lower 11C-PiB PET scan measurements of A*β* deposition as well as higher glucose metabolism as observed by FDG-PET scans [61, 62]. Higher MeDi adherence was also associated with reduced cerebral A*β* accumulation over time (up to 3 years) in a longitudinal study performed by Rainey-Smith et al. on older Australian adults classified as “A*β* accumulators” and thus considered to be on the way to AD [63]. However, the association between the Mediterranean dietary pattern and amyloid deposition or cortical thickness has not been confirmed in all reported studies. In a longitudinal study that involved 70 middle-aged participants living in New York, lower adherence to a Mediterranean-style diet was associated with faster decline in glucose metabolism in the posterior cingulate cortex (an early site of cerebral glucose utilization decline in AD and a well-established predictor of the progression from mild cognitive impairment to AD) and marginally in the frontal cortex, although without any significant changes in amyloid deposition or cortical thickness [64].

In conclusion, the neuroprotective effects of the MeDi may also lie in its ability to preserve brain metabolic activity and glucose metabolism in key brain regions for AD.

## 3. DASH Diet and AD Prevention

The DASH diet, which stands for Dietary Approaches to Stop Hypertension, promoted by the National Heart, Lung, and Blood Institute (NHLBI), is a dietary pattern that was first developed to identify dietary factors affecting blood pressure. The DASH diet is high in fruits, vegetables, nuts, whole-cereal products, low-fat dairy products, fish, and poultry, all of which are rich in blood pressure-deflating nutrients like potassium, calcium, “lean proteins,” minerals, and fiber (see Table 1). DASH also discourages the intake of foods like red and processed meats, full-fat dairy foods, and tropical oils, as well as sugar-sweetened beverages and sweets; therefore, it is designed to be low in sodium, saturated fats, total fats, and cholesterol [65]. This type of dietary pattern has been shown to protect against many cardiovascular risk factors that play a role in the development of dementia and AD (such as high blood pressure or LDL cholesterol), at least in part by modulating the pathological processes that characterize the physiopathology of AD (oxidative stress, inflammation, and insulin resistance) [66]. DASH and MeDi share many food components (i.e., whole-grains, vegetables, and nuts), but there are also some differences, such as the frequency of consumption of low-fat dairy products (moderate-to-high intake and low consumption, respectively).

To date, only a few studies have evaluated the potential effects of the DASH diet on cognitive functions or on the prevention of AD and other types of dementia.

### 3.1. Epidemiological Evidence

As already stated with regard to the Mediterranean diet, higher adherence to the DASH diet has been associated with slower rates of cognitive decline and reduced incidence of AD [67–69] (see Table 2).

In the study of Morris and colleagues [68], only the highest tertiles of the DASH and MeDi scores were significantly associated with a lower rate of incident AD (39% and 54% reduction, respectively).

Berendsen et al. [69] found a positive association between long-term adherence to the DASH diet and better cognitive function in older American women participating in the Nurses' Health Study, regardless of apolipoprotein E *ε*4 allele status, but not with slower cognitive decline during the course of follow-up (6 years). On the contrary, another study which included only older American women, the Women's Health Initiative Memory Study (WHIMS), reported that DASH scores were not associated with incidence of MCI or dementia [70].

### 3.2. RCTs

The only RCT which examined the potential effects of the DASH diet on neurocognitive functioning was performed some years ago by Smith and colleagues [71] (see Table 2). In the ENCORE trial 124 subjects with high blood pressure were randomized to the DASH diet alone or DASH combined with a behavioral weight management program including exercise and calorie restriction (CR), or to a “usual diet” control group. After 4 months of intervention, psychomotor function improved in both DASH interventions regardless of weight strategies, but only the group which underwent a combination of DASH diet with aerobic exercise and calorie restriction showed a significant improvement in neurocognitive function (executive memory and learning functions) [71].

## 4. MIND Diet and AD Prevention

The MIND diet (Mediterranean-DASH Intervention for Neurodegenerative Delay) was developed some years ago by researchers at Rush University Medical Center in Chicago, IL, and Harvard School of Public Health in Boston, MA, as a hybrid of the Mediterranean-DASH diet. The MIND diet is based on 10 brain-healthy foods (leafy green vegetables, other vegetables, nuts, berries, beans, whole grains, fish, poultry, olive oil, and wine) and 5 unhealthy foods (red meats, butter and stick margarines, cheese, pastries and sweets, and fried or fast food), all of which have a strong scientific rationale in the field of nutritional prevention of dementia [72, 73] (see Table 1). Compared to the MeDi, the MIND diet includes a lower consumption of fish, usually 1 serving/week, as does the DASH diet. This relatively low level of fish consumption reflects the findings of prospective epidemiological studies, such as the Rotterdam study which examined the role of fish intake in AD prevention [74–76]. As stated above, leafy green vegetables, in addition to other types of vegetables, are recommended on the basis of several epidemiological studies that reported a significant association between high consumption and slower cognitive decline [77, 78]. Berries represent a separate category in the MIND diet, reflecting strong associations between the consumption of this type of fruit and brain health. Many studies have documented the beneficial effects of various types of berries, including potent anti-inflammatory and antioxidant activity in cell models of neurotoxicity [79, 80]. Moreover, *in vivo* studies on animals supplemented with a berry-enriched diet reported improvements in motor coordination, cognitive performance (spatial memory, learning), hippocampal plasticity, and age-related neuronal loss [81–83]. A unique characteristic of berries is the high content of polyphenols such as flavonoids (anthocyanins and flavonols), which are the main compounds responsible for the antioxidant and anti-inflammatory characteristics of berries [84, 85]. The association between a high intake of berries and flavonoids and slower rates of cognitive decline have also been reported in humans [86], confirming the results that were observed in experimental studies.

### 4.1. Epidemiological Evidence

Two papers published by Morris and other researchers from Rush University (Chicago, IL, USA) clearly demonstrated the superiority of the MIND diet compared to both the MeDi and the DASH diet in slowing the rates of cognitive decline [28] and in reducing the risk of incident AD or dementia [68]. The MIND diet score was linearly and statistically significantly associated with a lower risk of developing AD (see Table 2). Subjects in both the middle and the highest tertiles of MIND scores had a statistically significant reduction in AD rates compared to those in the lowest tertile, while only those in the highest tertile of MeDi and DASH scores were significantly associated with a lower rate of incident AD [68]. To date, no randomized controlled trials have been published evaluating the effect of the MIND diet on the prevention of AD, but there are two ongoing studies testing the effects of an intervention with the MIND diet on cognitive decline and brain neurodegeneration, the results of which will be reported in the coming years (http://www.clinicaltrials.gov/NCT02817074, http://www.clinicaltrials.gov/NCT03585907).

## 5. Dietary Patterns and the Brain: The Underlying Mechanisms

The clinical evidence that has been collected in recent years suggests that the dietary patterns described above, namely, the Mediterranean diet, the DASH diet, and more recently the MIND diet, are able to modify the natural history of neurodegenerative pathologies, in particular AD, thereby preventing their development or slowing down their progression. But what is the basis of the favorable neuroprotective effects of these dietary patterns? What are the links between diet and the human brain? Are there any other factors that modulate the effects of diet on the brain?

The strength of these dietary approaches lies in their multifactorial composition. In fact, nutrient-dense foods can interact with each other with potentially synergistic effects on different metabolic and cellular signaling pathways, leading to neuroprotection and maintaining brain health. But there are considerable methodological difficulties in conducting *in vitro* and *in vivo* studies to document the effects of such dietary patterns on the brain, due to the intrinsic complexity of a whole diet, the micro- and macronutrient composition, the importance of food cooking methods, and at least for the Mediterranean diet, the undeniable aspect of conviviality that characterizes this dietary lifestyle. All of these aspects are obviously difficult to reproduce and quantify in experimental models.

To date, the precise mechanisms underlying the neuroprotective benefits associated with the MeDi and the other dietary patterns are not fully understood, even if it is widely accepted that they play a role in counteracting several biological processes implicated in the pathogenesis of AD, e.g., oxidative stress, neuroinflammation, neurovascular dysfunctions and hypoperfusion, disruption of the gut-brain axis, and impairment of hippocampal neurogenesis. It is also possible that these dietary patterns might influence A*β* or Tau metabolism, even if evidence concerning these mechanisms were largely obtained from animal studies and require further assessment and confirmation [87, 88]. At a systemic level, they can also indirectly improve cognition by reducing cardiovascular risk factors such as lipid levels, blood glucose, and blood pressure [89, 90].

### 5.1. Neurovascular Dysfunctions

Cerebral blood flow regulation is essential for normal brain function. AD is known to be associated with early neurovascular dysfunction and damage to small arteries, arterioles, and brain capillaries via both A*β*-independent (such as hypoxia and/or ischemia) and A*β*-dependent pathways (A*β* angiopathy). These changes, together with the loss of integrity of the blood-brain barrier (BBB), play a part in disease pathogenesis and lead to neuronal and synaptic dysfunction, neurodegeneration, and cognitive impairment [91, 92]. Several risk factors can contribute to neurovascular dysfunction, for example, genetic factors (APOE4 genotype), vascular factors (hypertension and diabetes mellitus), and environmental factors (for example, pollution). Hypertension is a well-known risk factor for AD, and animal studies have shown impairment in acetylcholine-dependent and endothelium-dependent vasodilation with a consequent reduction of cerebral blood flow after several stimuli [93]. Chronically elevated blood pressure can also enhance A*β*-induced neurovascular dysfunction, promote *β*-secretase activity, and lead to amyloidogenic processing of the amyloid precursor protein (APP), with further damage to small arteries and arterioles, rupture of the vessel wall, and microhemorrhages [93].

Hence, the importance and the possible preventive and neuroprotective role of a diet such as the DASH diet, which thanks to its reduced content of sugars, sweets/pastries, and sodium can act positively on some of the triggers of neurovascular dysregulation (hypertension and hyperglycemia/insulin resistance) [94]. In addition to limiting the intake of such detrimental foods and nutrients, increasing evidence supports the regular consumption of flavonoid-rich foods, which are associated with better endothelial function, tissue perfusion, and enhanced neuronal viability. For example, the flavonoids contained in cocoa powder (mainly epicatechin) act directly on the endothelium of brain vessels enhancing the activity of endothelial nitric oxide synthase (eNOS) and thereby increasing vasodilatation and cerebrovascular perfusion [95].

### 5.2. Oxidative Stress

The imbalance between the production of reactive oxygen species (ROS) and antioxidant defenses has been shown to contribute significantly to the pathogenesis and progression of AD [96] and is known to be associated with oxidative damage to DNA, proteins, and lipidic components of neurons and with subsequently impaired synaptic activity and neuronal apoptotic death [96]. Several antioxidant compounds can modulate signaling cascades involving protein and lipid kinases and downstream partners, resulting in the inhibition of neuronal apoptosis induced by toxic products such as ROS [97].

All three dietary patterns discussed in this review are exceptionally rich in foods with a high antioxidant content. Leafy green vegetables and other vegetables, citrus fruits, nuts, red wine, berries, and olive oil all provide a large amount of vitamins, polyphenols, and carotenoids that can prevent and/or repair the damage caused by free radicals (e.g., superoxide, O_2_^·−^ and nitric oxide, NO^·^) and by proinflammatory cytokines (e.g., IL-1*β* and TNF-*α*) that are produced by activated microglial cells in response to oxidative damage. One of the main components of the Mediterranean diet, as well as the main source of fat in this diet, is extravirgin olive oil (EVOO), which contains mainly oleic acid and to a lesser extent linoleic acid. In addition, EVOO contains hundreds of bioactive compounds including triterpenes (i.e., squalene), biophenols (hydroxytyrosol, tyrosol, and oleuropein), pigments (carotenoids, xanthophylls, and lutein), and vitamin E (tocopherols), whose beneficial effects have been shown in several studies [98, 99]. The low (and nontoxic) level of oxidized forms of EVOO polyphenols in plasma and tissues can activate the Nrf2 pathway and other adaptive stress response systems leading to the upregulation of the endogenous antioxidant and detoxification enzymes and thus rendering the cells “protected” against more dangerous and chronic oxidative stress stimuli (hormesis) [100]. Recent studies have shown that hydroxytyrosol, oleuropein, and oleacein can activate the Nrf2 pathway both *in vitro* and *in vivo* [101, 102].

### 5.3. Neuroinflammation

Microglial cells are the first and most important immune defense of the brain. While proper microglial function is essentially required for scavenging plaques, damaged molecules, and infectious agents, microglial hyperactivation is a well-established hallmark of neuroinflammation and one of the main actors in AD pathogenesis [103]. In the AD process, the excessive production and deposition of the A*β* peptides trigger an innate immune response and consequently an aberrant production of ROS, proinflammatory cytokine, and chemokine secretion, and degradation of the neuroprotective factors, such as retinoids, involved in promoting adult neurogenesis in the hippocampus [104, 105]. This chronic inflammatory response contributes to disease progression and severity, further boosts A*β* production and deposition, and ultimately leads to neuronal death [106, 107]. Several exogenous or endogenous factors can exacerbate the innate immune response mounted by A*β*-exposed microglia, including genetic factors (for example, TREM2 mutation [108] and APOE4 genotype [109]), traumatic brain injuries, diabetes [110], and obesity [111]. Diet is known to modulate the immune system, and a healthy diet rich in nutrients and bioactive compounds with anti-inflammatory and antioxidant properties can help to counteract the neuroinflammatory process.

Fruits, vegetables, whole grains, and other plant foods provide a wide range of phytochemicals, vitamins, minerals, and fibers with well-established antioxidant anti-inflammatory properties. Phytochemicals are bioactive plant-derived compounds that include various subgroups (phenolics, alkaloids, organosulfur compounds, phytosterols, and carotenoids). One example is Ferulic Acid (FA), an antioxidant with free radical scavenging activity but also antiamyloidogenic properties, as documented in a number of *in vitro* [112, 113] and *in vivo* studies [114, 115] in transgenic mouse models of AD. Resveratrol, another phenolic compound, is mainly found in the skin of many edible plant species, such as mulberries, grapes, peanuts, and pomegranates, as well as in red wine [116]. There are a multitude of actions that have been attributed to resveratrol: inhibition of Tau and A*β* plaque synthesis [117, 118], downregulation of prooxidative stress proteins [119] and increased levels of heme-oxygenase-1 (HO-1) [120] and SIRT-1, and inhibition of neuroinflammation [121, 122].

Long chain omega-3 polyunsaturated fatty acids (*n* − 3 PUFAs) are also important [123]. Docosahexaenoic acid (DHA) and Eicosapentaenoic Acid (EPA), two main types of *n* − 3 PUFAs, are abundantly present in seafood and fish oil. *In vitro* experiments showed that EPA and/or DHA administration decreases the expression of proinflammatory factors, such as inducible NO synthase (iNOS), cyclooxygenase (COX) 2, interleukin-1*β* (IL-1*β*), IL-6, Tumor Necrosis Factor-*α* (TNF-*α*), and Nuclear Factor-*κ*B (NF-*κ*B), and promotes the expression of anti-inflammatory cytokines. In rodents, the consumption of a diet enriched in *n* − 3 PUFAs prevents the dysregulation of cytokine production in hippocampal microglial cells in response to Lipopolysaccharide (LPS) [124], reduces hippocampal A*β* plaque density by modifying the fibrillar/prefibrillar A*β* oligomer ratio (the former are less toxic), and leads to mild improvements in the behavioral testing of the transgenic APP/PS1 rodent model of AD [125]. Moreover, dietary omega-3 and omega-6 PUFA and monounsaturated fatty acid (MUFA) intake may influence the membrane fluidity and enzyme activity in neurons, leading to the potential modulation of brain structures and functions [126].

### 5.4. Gut Microbiota Dysbiosis

The human gut microbiota includes approximately 10^14^ microbes belonging to hundreds different species and to five predominant phyla and is mainly composed of two phyla: *Firmicutes* (60-80%) and *Bacteroides* (20-30%), followed by *Actinobacteria*, *Verrucomicrobia*, and *Proteobacteria* [127, 128]. The gut microbiota plays a crucial role in human health but also in a variety of human diseases, at least in part through the production and release of numerous small molecules like vitamins (folate, vitamin B12), amino acids (tryptophan), and short chain fatty acids (SCFAs). Some of these bioactive substances (SCFAs, catecholamines, neurotransmitters, neuropeptides, and miRNAs) are transported into the blood and can cross the BBB thus affecting the central nervous system (CNS). Additionally, the CNS communicates with the gut through efferent autonomic pathways, thus modulating many gut functions like permeability, mucus secretion, motility, and immunity. This bidirectional communication between the gastrointestinal system and the CNS is called the gut-brain axis [129].

A healthy microbiota is fundamental for the metabolization of such dietary nutrients (like polyphenols) which require transformation to become active compounds having beneficial effects on the brain. Curcumin metabolites produced by the microbiota can exert anti-inflammatory and neuroprotective effects [130], including interesting positive effects on Tau pathology [131].

The composition of human gut microbiota is dynamic and can be shaped by various factors such as the type of childbirth and newborn feeding, diet, use of drugs, or pre/probiotics, as well as age, sex, and geographical area. The Mediterranean diet, which is rich in plant-based foods, fibers, and monounsaturated fats, is considered the gold standard for gut health and promotes the diversification of the microbiota [132]. Conversely, the typical Western diet, which is made up of low dietary fiber and high animal protein and saturated fat, is associated with a negative change in the gut microbiota composition (dysbiosis), as documented by an increase in Firmicutes and Gram-negative bacteria [133]. As previously mentioned, age is another important modulating factor of human gut microbiota composition. Several reviews analyzed gut microbiota age-related changes and the potential relationships between gut dysbiosis and inflammaging [134–136].

During aging, the gut undergoes a continuous and profound remodeling, as a result of modification of lifestyle, nutrition, behavior, immunosenescence (a decline in an immune system functioning), and inflammaging (the chronic low-grade inflammatory status typical of the elderly). Moreover, aging-associated alterations in gut physiology (i.e., gastric motility disorders, hypochlorhydria, and degenerative changes in the enteric nervous system) have profound effects on the diversity, composition, and functional features of intestinal microbiome [137]. Several authors reported a reduction in the microbiota diversity and a greater interindividual variation in microbiota composition in elderly people (>65 years of age), with reduced numbers of *Bifidobacteria*, *Firmicutes*, *Faecalibacterium prausnitzii*, and *Clostridium* cluster XIV and increased numbers of *Bacteroidetes* and *Enterobacteriaceae* even if with some differences between various populations [138–140].

The inflammatory process can affect the gut environment by enhancing the level of aerobiosis and the production of ROS that inactivate the strict anaerobic *Firmicutes*, while allowing the growth of facultative aerobes (i.e., *Enterobacteriaceae*, *Enterococcaceae*, and *Staphylococcaceae*). These so-called “pathobionts” can survive in the presence of oxygen, so they can prosper in an inflamed gut, and in turn, they promote a proinflammatory profile (increase of Il-6 and Il-8 levels) and compromise the host immune homeostasis, in a sort of self-sustaining loop [141].

Gut dysbiosis has been linked to the development of several health problems, including psychiatric or neurodegenerative diseases. An increasing body of evidence suggests that alterations to the gut microbiota can play a role in the pathogenesis of AD. Dysbiosis can amplify neuroinflammation and accelerate neurodegeneration, and this brain-gut microbiota axis can actually be modulated by dietary factors. In aging mice, AD-like symptoms were associated with increased gut permeability, inflammation, and a microbiome profile similar to that of murine inflammatory bowel disease [142]. Experimental mice models of AD showed a decrease in microbiota diversity with age, an increased number of taxa with proinflammatory activity (e.g., Odoribacter, Helicobacter, and Sutterella), and impaired production of SCFAs [143, 144]. All these modifications can be worsened by feeding transgenic mice a high-fat diet [145]. Moreover, gut microbiota dysbiosis in mouse models of dementia may be involved in neuroinflammation, reduced expression of hippocampal brain-derived neurotrophic factor (BDNF) and other signaling molecules, and amyloid deposition [146–148]. Conversely, administering probiotics to rodents with artificially induced AD led to an improvement in cognitive functions, especially spatial working tasks, less neuron degeneration and lower levels of proinflammatory cytokines [149–151], microglial activation and oxidative stress, improved mitochondrial dysfunction, and restoration of hippocampal plasticity [152].

In conclusion, long-term dietary habits may influence gut microbiota biodiversity, its functions, and the secretion of metabolites that, once absorbed in the systemic circulation, can modulate neural function and possibly enhance neuroinflammation, neuronal apoptosis, and *β*-amyloid deposition [153]. The modulation of gut microbiota by adopting and maintaining a healthy diet can be an effective strategy in AD prevention. However, the transposition of these results into humans is still premature given the absence of clinical studies, especially in middle-aged or older patients at risk of developing AD or with MCI.

### 5.5. Adult Hippocampal Neurogenesis

The hippocampus is the key brain region for learning and memory. Incorporation of new neurons into the granular cell layer of the dentate gyrus of the hippocampus is substantial throughout life, and adult neurogenesis has important implications in maintaining cognitive functions [154]. This brain area is highly involved in the process of neurodegeneration that is typical of AD. Numerous studies have documented an impairment of neurogenesis and relative memory loss as well as cognitive dysfunction in mouse models of AD even in the very early stages of the disease [155]. Environmental factors such as exercise and calorie restriction (see the specific chapter below) have been shown to increase adult hippocampal neurogenesis, while low-grade inflammation and oxidative stress seem to decrease it [156]. Hippocampal neurogenesis is regulated by several signaling pathways such as presenilin-1, Notch 1, soluble amyloid precursor protein, CREB, and *β*-catenin and is also mediated by neurotrophins such as BDNF. A poor diet can have detrimental effects on hippocampal neurogenesis. Diets that are rich in saturated fats/trans fatty acids and refined sugars, like Western diets, reduce the levels of BDNF and contribute to an increased production of ROS and proinflammatory cytokines, leading to neurodegeneration and learning and memory impairment [157]. On the contrary, dietary patterns based on foods that are rich in omega-3 fatty acids, flavonoids, and other antioxidants stimulate neurogenesis, reduce oxidative activity, and downregulate proinflammatory processes [158, 159].

## 6. Intermittent Fasting: A New Dietary Pattern for AD?

Aging is one of the main risk factors for AD. The aging brain and the “AD-Brain” share many characteristics, both at an anatomical and at a cellular/molecular level [160]. Indeed, many of the principal hallmarks of aging (e.g., oxidative stress, mitochondrial dysfunction, accumulation of oxidatively damaged molecules, impaired autophagy, disruption of Ca^2+^ homeostasis, aberrant neuronal network excitability, and neuroinflammation) have also been documented in AD, and these changes may promote amyloidogenic APP processing and Tau pathology and vice versa [161, 162].

Over the last 30 years, emerging evidence has shown the beneficial effects of fasting and CR as alternative or complementary strategies to other lifestyle interventions (e.g., physical activity) and to pharmacological therapies in AD prevention and treatment.

During the first 10-14 hours of fasting, the main source of energy for neurons is made up of glucose derived from the degradation of the liver glycogen store. Then, a “metabolic switch” occurs, characterized by liver production of Ketone Bodies (KBs) like *β*-hydroxybutyrate (BHB) and acetoacetate (AcAc) from the fatty acids released by adipose tissue in response to fasting; in this second phase, KBs represent the main fuel for neurons [163]. Besides this “metabolic switch,” fasting can enhance a complex series of adaptive responses to limited food availability, which are to some degree, the same endogenous stress-response systems activated by foods that are rich in polyphenols and other bioactive compounds (as described above) [164].

In prokaryotes, laboratory animals and humans, a daily 20-40% CR, can protect against aging, oxidative stress, and neurodegenerative disorders and can also extend longevity [165, 166], but the feasibility and long-term tolerability are low, especially in patients with AD. Other studies have shown the same positive results by alternating normal diets with more feasible regimens, such as intermittent fasting (IF) or periodic fasting (PF) that are short periods of fasting which differ from each other with regard to duration and frequency. The best-characterized form of fasting that has been studied both in animal models and in humans is Alternate-Day Fasting (ADF), which implies fasting every other day [167, 168] or a 70% CR every other day [169, 170] or even 2 consecutive days of Very Low-Calorie Diet (VLCD) per week [171]. Upcoming alternatives are the so-called Fasting-Mimicking Diets (FMDs), characterized by periodic cycles of plant-based dietary programs lasting from 3 to 7 days, that are low in protein and in overall caloric intake but contain all the necessary micronutrients [172, 173]. Regardless of how fasting has been applied in these studies, it should be emphasized that it is different from starvation, which leads to chronic insufficiency of nutrients, malnutrition, and ultimately degeneration and death.

### 6.1. Why Is Fasting Good for the Brain?

#### 6.1.1. Looking Briefly at the Molecular Mechanisms

**Figure 1:**
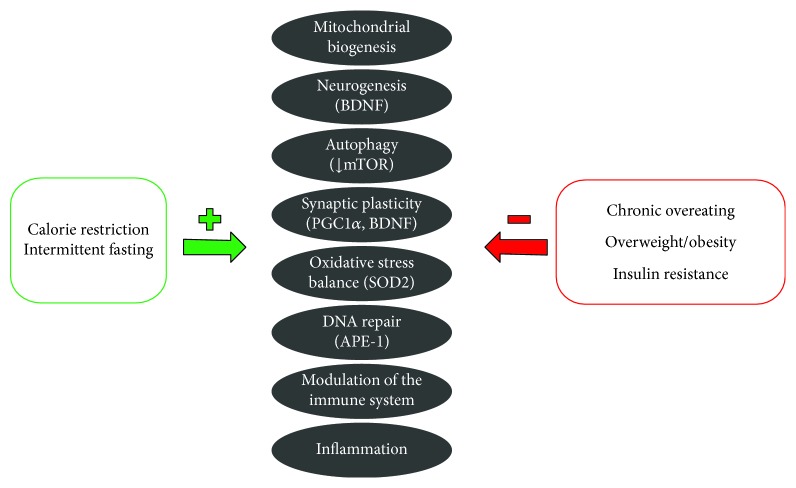
Signaling pathways involved in adaptive responses of neuronal circuits that contribute to maintain a healthy brain. Calorie restriction and intermittent fasting positively modulate these pathways, while chronic overfeeding and insulin resistance enhance neuroinflammation, neuronal damage, and apoptosis.

Fasting substantially modifies the neurochemistry and the activity of the neuronal network especially in several brain regions such as the hippocampus, the striatum, the hypothalamus, and the brainstem. At the molecular level, a variety of signaling pathways have been identified that mediate the structural (increased synaptic density, neurogenesis) and functional (Long-Term Potentiation, LTP) adaptive responses of neuronal circuits to nutrient restriction, in particular low glucose and amino acid levels [174] (see Figure 1). Both increased excitatory synaptic activity and neurotrophic factors (BDNF and fibroblast growth factor 2) lead to the activation of multiple kinases, including phosphatidylinositol 3-kinase (PI-3K), serine/threonine-protein kinase (AKT), Mitogen-Activated Protein Kinases (MAPKs) and Ca^2+^/calmodulin-dependent kinase (CaMK), nitric oxide synthase (NOS), and calcineurin, which all converge on several transcription factors like cAMP-Responsive Element-Binding protein (CREB), nuclear regulatory factor 2 (Nrf2), and NF-*κ*B [166, 175]. These transcription factors, which include BDNF [166], sirtuin-3 (SIRT3) [176], peroxisome proliferator-activated receptor *γ* coactivator 1*α* (PGC1*α*) [177], and heat-shock protein 70 (HSP-70), induce the expression of genes and proteins involved in enhancing neuroplasticity and stress resistance [178]. Part of the benefits of CR/fasting seems to be related to protein restriction and reduced IGF1/insulin signaling. During protein restriction, mTOR, and in particular complex 1 (mTORC1), is repressed, thus allowing the cell to enter a “conservative” energy mode to inhibit protein and lipid synthesis and enhanced autophagy [179, 180].

### 6.2. Fasting and AD

#### 6.2.1. Preclinical Studies

Most of the scientific evidence regarding IF or PF regimens and AD treatment derives from studies on laboratory animals. In rodents, several studies showed that fasting and FMDs are able to improve motor and cognitive functions, in particular hippocampal-dependent tasks like learning and memory [172, 181–183]. Moreover, IF has been associated with reduced oxidative stress and brain structural improvements such as increased thickness of the CA1 pyramidal cell layer and higher expression of the dendritic protein drebrin in the hippocampus [184].

IF and ketogenic diets can also modify neuronal network activity and synaptic plasticity. The neurons of rodents on an ADF regimen are more resistant to excitotoxin-induced degeneration of hippocampal neurons with kainic acid and perform better in water-maze learning and memory tasks [167, 185]. IF and/or regular physical activity can also prevent age-related deficits in LTP, a common cellular manifestation of learning and memory occurring in response to repetitive stimulation of synapses [186–189]. Other research groups also documented a role of CR/IF regimens in reducing A*β* deposition and Tau phosphorylation in the hippocampus and cerebral cortex of a transgenic mouse model of AD [190–194]. Recently, Zhang et al. highlighted the role of IF (and in particular BHB) in restoring the polarity of AQP4, a protein channel involved in A*β* clearance, that is often impaired in AD, thereby providing another possible explanation for the positive role of ADF in improving cognitive functions and protecting against A*β* pathology [195].

Lastly, IF, with or without exercise, stimulates the growth and differentiation of new neurons into granule neurons in the hippocampal dentate gyrus, i.e., adult hippocampal neurogenesis, and the creation of synaptic connections (dendritic spine growth and synaptogenesis) among themselves or with other neurons from other brain regions such as the entorhinal cortex, basal forebrain, and amygdala [172, 196, 197].

#### 6.2.2. CR and IF in Humans

Several human studies have shown that the decrease in energy intake can reduce visceral fat (while preserving lean mass), improve glucose and lipid metabolism, and reduce blood pressure and blood biomarkers of inflammation (C-reactive protein and proinflammatory cytokines) [171, 198, 199]. All these beneficial effects may translate into a lower risk of cardiovascular disease, diabetes, and also neurodegenerative diseases such as AD.

Currently, no studies have been conducted on protein and/or calorie restriction in human subjects with established AD, but some authors have underlined the potential role of protein restriction against the aging process and aging-related chronic diseases [200]. However, it is important to properly time the application of protein restriction during life since the beneficial effects seem to be lost in people over 65 years of age [201].

Short periods of CR are able to improve cognitive function (verbal memory) in elderly subjects [202], and 30 days of a low glycemic diet in patients with MCI resulted in an improvement in delayed visual memory, cerebrospinal fluid biomarkers of A*β* metabolism, and brain bioenergetics [203], but it is hard to believe that severe restrictions could be tolerated for long periods, especially in elderly subjects affected by neurodegenerative diseases. IF regimens or FMD cycles every 1-2 months seem to be more feasible and tolerable in clinical practice. Recent pilot clinical trials applying such dietary regimens in healthy subjects and in patients with cancer, diabetes, and multiple sclerosis have been developed and have shown promising results [172, 173, 204], while several trials are still ongoing (https://clinicaltrials.gov/NCT03595540, https://clinicaltrials.gov/NCT03700437, and https://clinicaltrials.gov/NCT03811587).

The next step will be to demonstrate whether the various types of fasting (including ADF and FMD) have neuroprotective and regenerative effects in patients with early-stage AD. Our group will carry out a pilot study in an effort to assess the safety and feasibility of monthly cycles of an FMD diet in patients with a diagnosis of MCI or early AD.

In conclusion, emerging evidence on the effects of fasting on animal models of aging brain and neurodegenerative diseases is promising, but the applicability and potential efficacy of these dietary regimens in humans, and in particular in patients with MCI or AD, are yet to be established.

## 7. Conclusions

To date, it is not possible to establish with certainty a causal relationship between diet and the development of AD because there are still many confounding factors and biases:

1. There may be confounding factors that contribute to increasing or reducing the risk of AD (physical activity, cardiovascular risk factors, and apoE4 status)
2. There is broad heterogeneity among the characteristics of the studies, such as the mean age of the study subjects (50-85 years), diverse geographic settings (differences in the dietary patterns or in the environmental factors in Mediterranean or non-Mediterranean regions), sample size, study designs (cross-sectional, prospective, case-control, and RCTs), and length of follow-up
3. Several different methods have been used for evaluating eating habits (different questionnaires, food diary, and 24-hour dietary recall)
4. There is broad heterogeneity in the criteria used for the evaluation of cognitive performance and diagnosis of AD (single neuropsychological tests or neuropsychological batteries)
5. None of the studies take into account the possible modifications in an individual's eating habits over the course of one's life as related to psychological (e.g., depression), physiological (e.g., difficulty in chewing and modification of appetite), or socioeconomic changes (e.g., social isolation, financial difficulties, and lack of family support)
6. There is broad heterogeneity in the study outcomes (AD incidence or prevalence, worsening of the overall cognitive performance or even of some specific cognitive domains)

Currently available drugs (cholinesterase inhibitors and memantine) are able to partially control the symptoms but do not slow down the progression of AD. Therefore, there is an urgent need for new complementary therapeutic approaches, and in this context, the modulation of dietary habits and well-conducted nutritional interventions could be a useful and inexpensive tool.

## Figures and Tables

**Table 1:** Type and frequency of consumption of foods of the various dietary patterns having a role in AD prevention.

Dietary pattern	Characteristics	
	Moderate-to-high consumption	Low consumption
Mediterranean diet (MeDi)	Whole-grain cereals (1-2 s every main meal) Vegetables (≥2 s/every main meal) Fruits (1-2 s/every main meal) Olive oil (every main meal) Olives/nuts/seeds (1-2 s/every day) Low-fat diaries (2 s/every day) Herbs/spices/garlic/onions (every day) Eggs (2-4 s/week) White meat (2 s/week) Fish/seafood (≥2 s/week) Potatoes (≤3 s/week) Legumes (≥2 s/week) (Red) wine	Added salt Red meat (<2 s/week) Processed meat (<1 s/week) Sweets (≤2 s/week)

Dietary Approaches to Stop Hypertension (DASH)	Whole-grain products (every day) Vegetables (every day) Fruits (every day) Diary (moderate-to-high consumption) Poultry (2 s/week) Fish/seafood (1-2 s/week) Legumes (2 s/week)	Saturated fats Total fats Salt (sodium) Sweetened beverages Red and processed meats

Mediterranean-DASH Intervention for Neurodegenerative Delay (MIND)	Green leafy vegetables (≥6 s/week) Other vegetables (≥1 s/day) Nuts (≥5 s/week) Berries (≥2 s/week) Beans (≥3 s/week) Whole grains (≥3 s/day) Fish (≥1 s/week) Poultry (≥2 s/week) Olive oil (primary oil used) (Red) wine (1 glass/day)	Red meats (<4 s/week) Butter and stick margarines Cheese (<1 s/week) Pastries and sweets (<5 s/week) Fried or fast food (<1 s/week)

**Table 2:** Summary of the clinical studies that investigate the role of the three dietary patterns (MeDi, DASH, and MIND) in counteracting cognitive decline, incidence of dementia, and/or AD and AD-related mortality.

Authors (year)	Type of diet	Study design	Study population	Results	Follow-up/length of intervention	Reference
Scarmeas et al. (2006)	MeDi	Cross-sectional	Elderly Americans (NY)	Higher adherence to the MeDi was associated with lower risk for AD (odds ratio, 0.76; 95% confidence interval, 0.67-0.87; P.001)	NA	[22]
Gardener et al. (2012)	MeDi	Cross-sectional (AIBL) study	Elderly Australians	Compared with healthy controls, subjects with AD and MCI had a lower mean MeDi score (*P* < 0.001 and <0.05, respectively); each additional unit in the MeDi score was associated with 13–19% lower odds of being in the MCI category, and 19–26% lower odds of being in the AD category	NA	[23]
Scarmeas et al. (2006b)	MeDi	Cohort	Elderly Americans (NY)	Higher adherence to the MeDi was associated with significantly lower risk for development of AD. Each additional unit of the MeDi score was associated with 9 to 10% less risk for development of AD	4 years	[25]
Scarmeas et al. (2009)	MeDi	Cohort	Elderly Americans (NY)	Both higher Mediterranean-type diet adherence and higher physical activity were independently associated with reduced risk for AD	5.4 years	[26]
Gu et al. (2010)	MeDi	Cohort	Elderly Americans (NY)	Significant association between MeDi score and reduction in risk of AD: compared to those in the lowest tertile of MeDi, subjects in the highest tertile had a 34% less risk of developing AD (p‐for‐trend = 0.04)	3.8 years	[27]
Morris et al. (2015)	MeDi DASH MIND	Cohort	Elderly Americans (Chicago)	Participants in both the middle and the highest tertiles of MIND scores had a statistically significant reduction in AD rate compared to those in the lowest tertile (53% and 35% reduction, respectively). Subjects with the highest adherence to the MeDi and DASH had a 54% and 39% lower risk of developing AD, respectively, compared to those in the lowest tertile (HR = 0.46, 95% CI 0.26, 0.79)	4.5 years	[28]
Scarmeas et al. (2009b)	MeDi	Cohort	Elderly Americans (NY)	Significant association between MeDi adherence and MCI conversion to AD, with a 48% less risk of developing AD (HR: 0.52; 95% CI: 0.30, 0.91; *P* = 0.02) for highest vs. lowest tertile on MeDi score	4.3 years	[29]
Scarmeas et al. (2007)	MeDi	Cohort	Elderly Americans (NY)	Higher adherence to the MeDi is associated with lower mortality in AD patients	4.4 years	[30]
Anastasiou et al. (2017)	MeDi	Cross-sectional	Elderly Greeks	Adherence to MeDi is positively associated with a decreased likelihood of dementia and better cognitive performance in many domains, especially memory	NA	[31]
Martinez-Lapiscina et al. (2013)	MeDi	RCT (PREDIMED)	Individuals at high CV risk (from Spain)	A dietary intervention with MeDi enriched with either EVOO or nuts appears to improve cognition compared with a low-fat diet	6.5 years	[43]
Valls-Pedret et al. (2015)	MeDi	RCT (PREDIMED)	Individuals at a high CV risk (from Spain)	A MeDi supplemented with EVOO or nuts is associated with improved composite measures of cognitive function	4.1 years	[44]
Knight et al. (2016)	MeDi	RCT (MedLey)	Elderly Australians	No evidence of a beneficial effect of a MeDi intervention on cognitive function among healthy older adults	6 months	[45]
Marseglia et al. (2018)	MeDi	RCT (NU-AGE)	Five European populations	Improved cognitive performance in both the active and the control groups, with no additional diet-related cognitive improvements	1 year	[46]
Tangney et al. (2014)	MeDi DASH	Cohort	Older Americans (Chicago)	A 1-unit difference in DASH score and in MedDietScore are associated with a slower rate of global cognitive decline by 0.007 standardized units (standard error of estimate = 0.003, *P* = 0.03) and by 0.002 standardized units (standard error of estimate = 0.001, *P* = 0.01), respectively	4.1 years	[67]
Berendsen et al. (2017)	DASH	Cohort	Older American women	Long-term adherence to the DASH diet is associated with better average cognitive function but not with change in cognitive function over the follow-up period	6 years	[69]
Haring et al. (2016)	MeDi DASH	Cohort	Older American women	No association between aMED and DASH scores and incidence of MCI or dementia in older women generally or in those with hypertension	9.1 years	[70]
Smith et al. (2010)	DASH	RCT (ENCORE)	Overweight and sedentary individuals (USA)	Slight improvements in psychomotor speed after the intervention with the DASH diet	4 months	[71]
McEvoy CT et al. (2017)	MeDi MIND	Cross-sectional	Older U.S. adults	Greater adherence to the MeDi and MIND diet is independently associated with better cognitive function and lower risk of cognitive impairment	NA	J Am Geriatr Soc. (2017) 65:1857–1862
